# Understanding practices of lactation and infant feeding together with women with HIV: the UPLIFT observational study protocol

**DOI:** 10.3389/frph.2026.1856899

**Published:** 2026-07-08

**Authors:** Christiana Smith, Emily Anne Barr, Karen Hampanda, Mark Trumbull, Hulin Wu, Jennifer McKinney, Sarah Buisson, Carly Hoffmann, Ciarra Brown, Angela B. Hutchinson, K. Rivet Amico, Ifeyinwa V. Asiodu, Elizabeth J. McFarland, Margaret A. Lampe, Preetam A. Cholli, Olga Varechtchouk, Allahna Esber, Athena P. Kourtis, Lisa Abuogi

**Affiliations:** 1 Department of Pediatrics, Section of Infectious Diseases and Epidemiology, School of Medicine, University of Colorado Anschutz Medical Campus, Aurora, CO, United States; 2 School of Nursing, University at Buffalo, Buffalo, NY, United States; 3 Department of Obstetrics and Gynecology, School of Medicine, University of Colorado Anschutz Medical Campus, Aurora, CO, United States; 4 Frontier Science & Technology Research Foundation, Amherst, NY, United States; 5 Department of Biostatistics & Data Science, School of Public Health, University of Texas Health Science Center at Houston, Houston, TX, United States; 6 Department of Obstetrics and Gynecology, Division of Maternal Fetal Medicine, Baylor College of Medicine, Houston, TX, United States; 7 FHI 360, Durham, NC, United States; 8 The Well Project, Brooklyn, NY, United States; 9 Division of HIV Prevention, National Center for HIV, Hepatitis, Sexually Transmitted Diseases and Tuberculosis Prevention, Centers for Disease Control and Prevention (CDC), Department of Health and Human Services (HHS), Atlanta, GA, United States; 10 University of Michigan, School of Public Health, Ann Arbor, MI, United States; 11 School of Nursing, University of California San Francisco, San Francisco, CA, United States; 12 Division of AIDS, National Institute of Allergy and Infectious Diseases, National Institutes of Health, Rockville, MD, United States; 13 Maternal and Pediatric Infectious Disease Branch, Eunice Kennedy Shriver National Institute of Child Health and Human Development, Bethesda, MD, United States

**Keywords:** breast milk, breastfeeding, infant exposure to HIV, infant feeding, lactation, perinatal HIV transmission, pregnancy, woman living with HIV

## Abstract

**Introduction:**

There is a limited understanding of infant feeding decisions, practices, and outcomes for women living with HIV (WLHIV) and their infants since U.S. guidelines were updated in 2023 to support breastfeeding for virally suppressed women on antiretroviral treatment.

**Methods and analysis:**

The Understanding Practices of Lactation and Infant Feeding Together with women with HIV (UPLIFT) study is a convergent mixed-methods, multisite, observational study designed to address key knowledge gaps. Twelve US sites will begin enrollment in early 2026 to accomplish three core activities (CA). In CA1, we explore infant feeding decision-making and experiences via in-depth interviews with pregnant/postpartum WLHIV (*n* = 50), their partners and other influential individuals (*n* = 25), and a diverse sample of healthcare professionals (*n* = 45). Site-level surveys assess infant feeding practices and estimate the prevalence of breastfeeding among WLHIV. In CA2, we establish an observational longitudinal cohort of 450 WLHIV-infant dyads to examine infant feeding practices and outcomes. WLHIV will be enrolled during pregnancy and early postpartum, with half intending to breastfeed, and will be followed for ≥48 weeks. Clinical and laboratory data will be collected. Comprehensive surveys collect data on demographics, social determinants of health, mental health, antiretroviral therapy adherence, and infant feeding intentions and experiences. We measure HIV RNA and DNA in breast milk to determine factors associated with detectable HIV. Finally, we estimate the costs and benefits associated with breastfeeding compared to replacement feeding. In CA3, we develop and pilot a voluntary registry of U.S. WLHIV who are breastfeeding and their infants (*n* = 50−200). Pilot registry testers (*n* = 30) will provide feedback on the usability, acceptability, appropriateness, and feasibility of the registry.

**Discussion:**

The UPLIFT study will generate critical insights to inform patient-centered care, guide practice and policy, and empower WLHIV in the US to make informed infant feeding decisions.

**Ethics and dissemination:**

The UPLIFT study received approval from Advarra, the institutional review board of record for all UPLIFT sites. Results will be disseminated through conference presentations, peer-reviewed journals, and lay summaries.

**Clinical Trial Registration:**

https://clinicaltrials.gov/study/NCT07293559, NCT07293559.

## 1 Introduction

The landscape of infant feeding among women living with HIV (WLHIV) in the United States (U.S.) has undergone a dramatic shift in recent years. Prior to 2023, breastfeeding while living with HIV was contraindicated in the U.S (1). WLHIV were discouraged from breastfeeding due to concerns that the risk of HIV transmission outweighed any potential benefits to the mother or infant (2). However, cumulative research has demonstrated very low rates (<1%) of HIV transmission via breastfeeding among virologically suppressed women on antiretroviral therapy (ART) (3–10). These data, combined with recognition of the long-term health benefits of breastfeeding and increasing advocacy from WLHIV, healthcare professionals, and key stakeholders to center autonomy, health equity, and cultural considerations in infant feeding decisions, led multiple U.S. advisory groups, including the Department of Health and Human Services (HHS) and the American Academy of Pediatrics, to update their guidelines to recommend an evidence-based, family-centered, shared decision-making approach to infant feeding discussions (11, 12).

Approximately 3,300 WLHIV give birth each year in the U.S. (13–15). U.S. WLHIV have increasingly expressed a desire to breastfeed, citing numerous potential benefits such as maternal-infant bonding, alignment with cultural and social norms, and the nutritional and immune benefits of breast milk (16–20). Breastfeeding is protective against infections, allergic conditions, and chronic diseases such as obesity in infants, and reduces the likelihood of postpartum depression, breast and ovarian cancer, hypertension, and type 2 diabetes in mothers (21, 22). When WLHIV choose not to breastfeed, it may inadvertently disclose their HIV status, impose a sense of stigma, and/or affect adverse mental health outcomes (23–27). Moreover, replacement feeding (i.e., alternatives to breastmilk, such as formula) can be cost-prohibitive and recent formula shortages and recalls in the U.S. have demonstrated the potential for access issues, with significant accompanying tangible and intangible costs for parents (28–30). Recent qualitative studies reveal that WLHIV in the U.S. weigh numerous motivations for their infant feeding decisions, with HIV transmission risk representing just one of several factors considered (31, 32).

Most data on breastfeeding transmission risk are derived from cohort studies conducted in low- and middle-income countries (LMIC), where breastfeeding has been encouraged among WLHIV for decades (33, 34). These studies demonstrate that infant HIV transmission during breastfeeding is substantially reduced, but not eliminated, by receipt of ART in WLHIV and/or antiretroviral (ARV) prophylaxis in infants (3–10). The PROMISE study described a 0.6% transmission rate by 12 months of age among breastfeeding infants randomized to either maternal ART or prolonged infant nevirapine prophylaxis (6). Subsequent cohort studies have described breastfeeding transmission rates ranging from 0%–0.8%, with some studies reporting lower transmission rates associated with frequent maternal viral load monitoring and with the availability of infant prophylaxis and/or free infant replacement foods in the event of maternal viral load elevation (5, 7–9). Despite these successes, there were several reported cases of transmission to infants via breastfeeding. Instances of HIV diagnoses among infants of mothers receiving ART with undetectable plasma viral loads suggests that the concept of “U = U” (undetectable = untransmittable)—while established for sexual HIV transmission—may not be applicable to breastfeeding transmission (5–7, 35–42).. Recent meta-analyses have estimated a monthly risk of breastfeeding transmission of 0.1% when the most recent maternal viral load is <50 copies/mL and 0.02% when ART is initiated prior to conception (43, 44). To our knowledge, no breastfeeding transmission events have been reported among WLHIV who both initiated ART prior to conception and maintained an undetectable viral load throughout pregnancy and breastfeeding.

Many gaps still exist in our understanding of how HIV transmission risk during breastfeeding applies to the U.S., where WLHIV and their infants may benefit from increased access to ART and infant prophylaxis, virologic monitoring, and infant replacement foods (45). Adherence to ART in the postpartum period is a recognized challenge for WLHIV regardless of geographic setting (46–48). Additionally, WLHIV in the U.S. frequently experience lapses in HIV care engagement postpartum due to insurance changes and transitions to long-term adult HIV care providers (49, 50). Several clinical scenarios have traditionally been associated with an increased risk of transmission during breastfeeding - certain breast conditions (e.g., mastitis); oral lesions in the infant (e.g., thrush); mixed feeding (providing a combination of breast milk, formula, and/or other foods); and abrupt weaning. However, none of these factors have been systematically and prospectively studied in a population of WLHIV in the U.S. who are virologically suppressed on ART (51–60).

The shift towards a shared decision-making model for infant feeding among WLHIV has numerous practical and ethical implications (61). Healthcare providers (HCPs) who counsel WLHIV may feel anxiety or discomfort around this topic, especially if they are not well-versed in the data that informed the recent U.S. guideline changes. A 2023 survey of U.S. Pediatric Infectious Diseases Society (PIDS) members highlighted significant heterogeneity in the uptake of the updated HHS infant feeding guidelines, with 20% reporting either being unaware of the guidelines change or having no plans to implement them (62). A subsequent survey of PIDS members and neonatologists in 2024 identified that fewer than half of respondents would offer breast milk from a virally suppressed parent with HIV as an infant feeding option, with substantial geographic variability (63). Importantly, these studies also demonstrated that a minority of U.S. institutions have established guidelines or policies for feeding breast milk from a parent with HIV and identified wide variability in institutional practices surrounding infant antiretroviral prophylaxis, viral load monitoring for mothers and infants, and approaches to mixed feeding. This practice variability has also been demonstrated in several published case series of WLHIV who have breastfed in the U.S (64–68).

Thus far, U.S. case series have reported only a single episode of infant HIV acquisition during breastfeeding in a mother-infant dyad that was lost to follow up with maternal discontinuation of ART (64–68). However, these publications are insufficient to establish generalizable findings on the epidemiology, management, or outcomes of U.S. WLHIV who choose to breastfeed. U.S. HCPs have emphasized the need for additional data from a large sample size of breastfeeding WLHIV in high-income countries to increase their comfort with supporting breastfeeding in this population (62). Some publications have specifically called for the establishment of a centralized registry to aggregate data on breastfeeding outcomes from WLHIV and their infants across multiple U.S. sites (63).

To address these scientific gaps, the Understanding Practices of Lactation and Infant Feeding Together with women with HIV (UPLIFT) study was developed (69) with funding from the Centers for Disease Control and Prevention (CDC) and National Institutes of Health (NIH) with support from the International Maternal Pediatric Adolescent AIDS Clinical Trial (IMPAACT) Network. UPLIFT opened for enrollment at 12 U.S. sites in February 2026 (NCT07293559) (70). In this manuscript, we present the UPLIFT study protocol, including the objectives, design, and methods that will be employed. This manuscript uses the terms “woman/women,” “mother,” and “breastfeeding;” however, we acknowledge that some individuals may use alternative terminology and we encourage providers to use the language preferred by the individuals receiving care.

## 2 Methods and analysis

### 2.1 Overall study design

**Figure 1:**
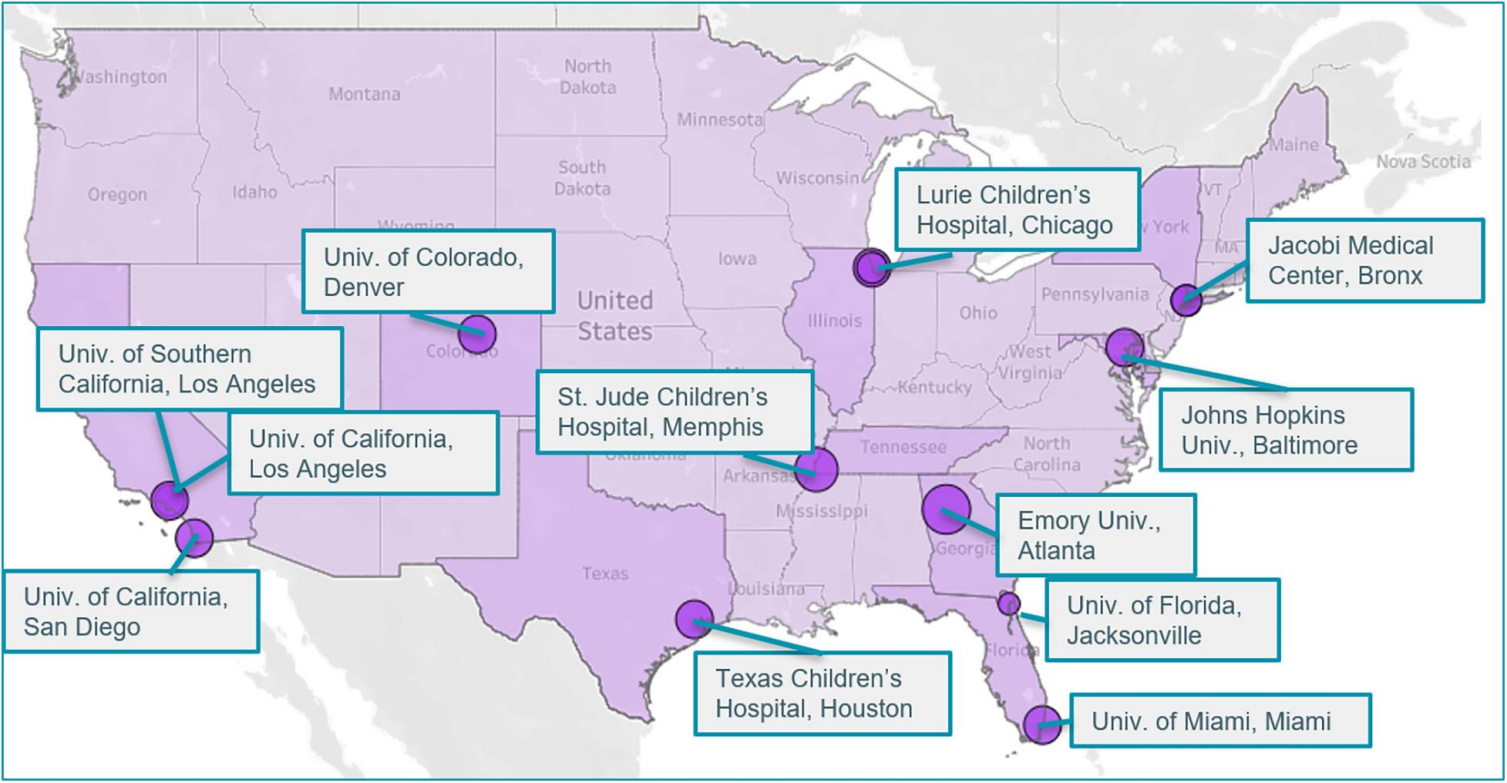
Map of UPLIFT study sites. Twelve U.S. sites are depicted with the associated institution and location.

**Figure 2:**
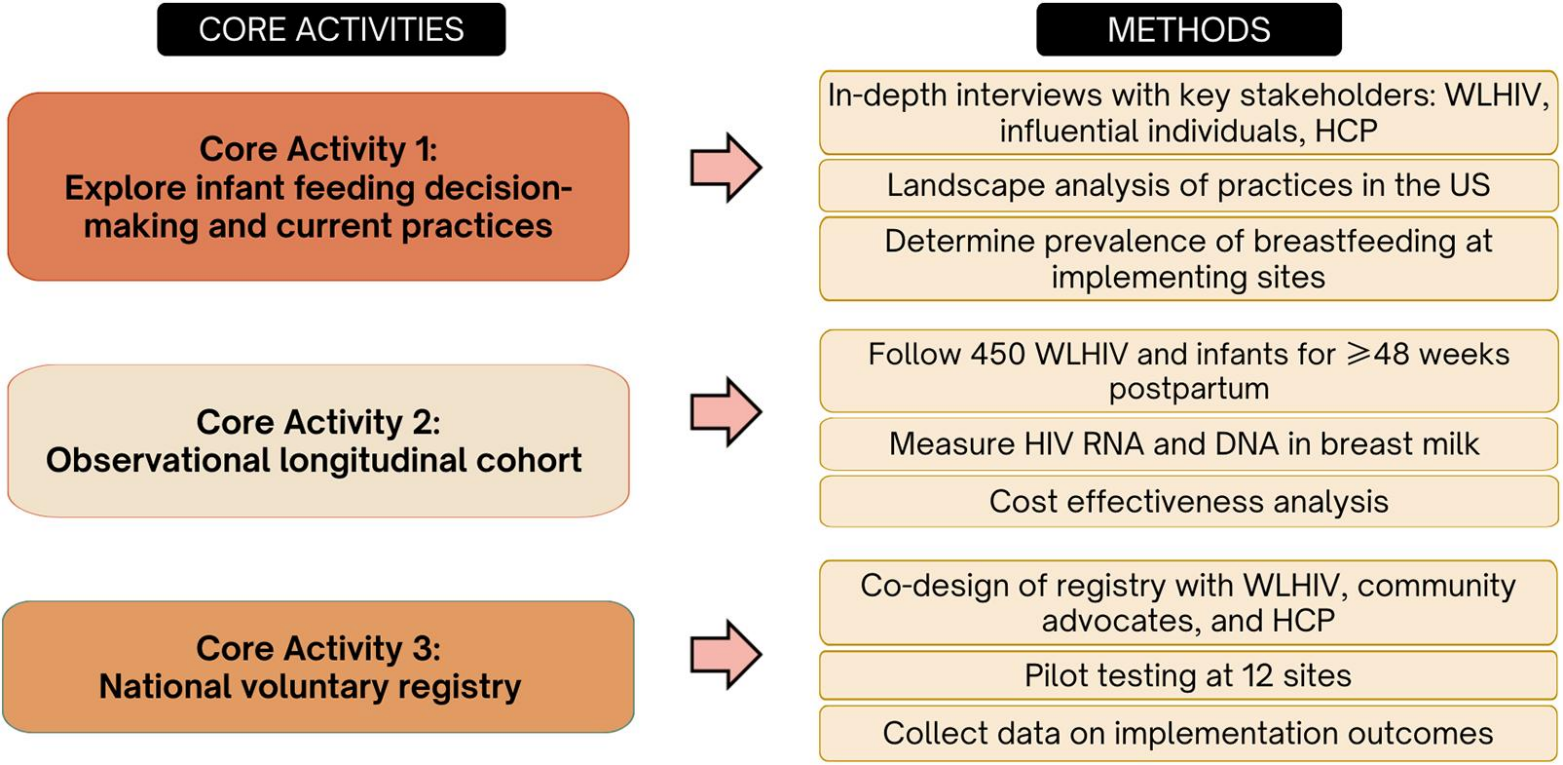
Overview of three core activities. Each Core Activity is described along with associated methods. HCP, Healthcare Professionals; US, United States; WLHIV, women living with HIV.

UPLIFT is a convergent mixed-methods observational study that will enroll participants from 12 geographically diverse sites across the U.S. (Figure 1). UPLIFT will leverage the infrastructure of the International Maternal, Pediatric, Adolescent AIDS Clinical Trials (IMPAACT) Network, an international multisite research network that conducts longitudinal studies of infants, children, adolescents, pregnant and postpartum women who are living with or impacted by HIV (71). UPLIFT will achieve its scientific objectives through three overarching core activities (CA; Figure 2). Core Activity 1 will conduct in-depth interviews with key stakeholders to determine how infant feeding decisions among WLHIV in the U.S. are influenced by factors at individual, interpersonal, health system, and socio-cultural levels. Core Activity 1 will also estimate the site-level prevalence of breastfeeding among WLHIV and determine current site-level practices and standards of care for the management of infant feeding among WLHIV. CA2 will enroll a prospective observational cohort of WLHIV during pregnancy and postpartum to determine the demographic, psychosocial, and clinical factors that influence infant feeding decisions and the infant feeding preferences, practices, and outcomes of U.S. WLHIV and their infants. Core Activity 3 will develop and pilot a voluntary registry designed to identify epidemiological trends among breastfeeding WLHIV-infant dyads in the U.S. In the UPLIFT study, breastfeeding will be defined as any infant exposure to breast milk from a WLHIV. This definition will include direct feeding from the breast, feeding of expressed milk, and mixed breast milk and formula feeding. Replacement feeding will be defined as no exposure to breast milk from the mother with HIV, which could include feeding with infant formula, donor breast milk, solid foods, or any other replacement food.

#### 2.1.1 Recruitment, enrollment, and retention

Recruitment methods will primarily rely upon outreach to current patients being followed for clinical indications at study sites or active identification and referral of patients from affiliated institutions. Informed consent will be obtained by trained study staff designated by the investigator of record at each site. WLHIV may co-enroll into CA1, 2, and/or 3, if eligible. Accrual will be monitored by the protocol team on a monthly basis, who will address barriers to enrollment as needed. After enrollment, study staff will make every effort to retain participants in follow up for the protocol-specified duration, thereby minimizing potential biases and loss of statistical power. Retention strategies will vary by site but may include strategies such as frequent contact between study visits. Participants will be compensated for costs associated with study visits such as transportation costs, based on local standards of care.

#### 2.1.2 Data management

Frontier Science and Technology Research Foundation, Inc. (Frontier Science) will serve as the Data Management Center (DMC) for this study, providing the systems for data collection as well as registry design. Data collection tools used for the UPLIFT study will include Medidata's Clinical Data Management platform, Medidata Rave, already in use at IMPAACT sites; Medidata's electronic patient-recorded outcome (ePRO) application, Patient Cloud; and REDCap hosted by Frontier Science. Enrolled participants will be assigned a coded Participant Identifier (PID) to minimize collection of personally identifiable information as required for the study and to ensure data confidentiality before, during, and after the study. Any data disseminated from the study will be shared using the PID and vetted through IMPAACT data request procedures. All data will be stored in the IMPAACT central database at Frontier Science's production computing site, a secure, SOC II certified data center located in Albany, NY. The DMC ensures that all systems comply with privacy, security, and accessibility requirements such as the Section 508 amendment to the Rehabilitation Act, 21 Code of Federal Rulings (CFR) Part II, and the security control framework of the Federal Information Security Management Act of 2002 (FISMA) as documented in the National Institute of Standards and Technology Special Publication 800−53.

During the study, the Protocol Data Manager will oversee quality control and quality assurance of all clinical data submitted to Frontier Science, while the Laboratory Data Manager is responsible for quality control and quality assurance of the biospecimen and laboratory data collected. Each role performs rigorous data quality checks based on risk as outlined in the Data Management Plan (DMP) and Integrated Data Reviewers Guide (IDRP). Automated edit checks are programmed into the Medidata Rave Electronic Data Capture system and alert when there are missing or out of range entries. In addition, data managers and medical coders review all submitted data and issue manual queries as needed to the submitting site or laboratory. Built-in task management and data cleaning dashboards within the Medidata platform alert the site regarding data that is overdue, non-conformant, or with outstanding queries.

#### 2.1.3 Study monitoring

The protocol team will monitor key indicators of study quality, including protocol deviations, data quality, and data completeness, based on monthly reports from the DMC. Participant clinical events of interest (e.g., hospitalizations) will be reviewed monthly by the protocol team and assessed for any concerning trends. A subset of clinical events will be reported to the study team within 7 days (e.g., infant HIV acquisitions, participant deaths). Any concerning participant responses identified on surveys or interactions with study team members (e.g., suicidal risk, intimate partner violence) will trigger real-time notifications to site staff who are expected to manage these concerns according to local standard operating procedures. Study oversight will be provided by the IMPAACT Management Oversight Group (MOG), who will review the monthly study operations report. An independent IMPAACT Study Monitoring Committee (SMC) provided initial protocol review before the study opened to enrollment, and will provide *ad hoc* reviews if any concerns arise from the protocol team or MOG. Any protocol modifications after study opening will require approval from sponsoring institutions, key members of the protocol team, and the institutional review board. Modifications will be communicated to the MOG and to site staff through site training.

#### 2.1.4 Community engagement

Community engagement is foundational to the UPLIFT study. The study team includes several core members who are WLHIV who have breastfed and/or supported other WLHIV in their infant feeding decision-making. These community members are integrated into the research team and are actively engaged across study phases. In addition, UPLIFT partners with The Well Project, a national nonprofit organization whose mission is to change the course of the HIV/AIDS pandemic through a unique and comprehensive focus on women and girls, as consultants and significant contributors throughout study design and implementation (72). As needed, members of the IMPAACT Community Advisory Board are consulted to provide broader community input.

Core community stakeholders contributed substantively to study development, including reviewing and refining protocol language, co-developing and reviewing in-depth interview guides, and providing extensive feedback on study questionnaires. Additionally, community members will pilot instruments and participate in mock interviews to support interviewer training and preparedness. Community contributors also informed website design, participated in site trainings focused on bias, language use, and trust-building, will serve as members of the qualitative analysis team, where they will support the development and refinement of analytic codes and themes. Finally, community stakeholders will play a central role in results interpretation and dissemination, supporting participant-centered and accessible approaches that prioritize transparency, respect, and trust.

### 2.2 Core Activity 1 (CA1)

#### 2.2.1 Core Activity 1 objectives

The primary and secondary objectives of CA1 are presented in Table 1.

**Table 1:** Objectives of Core Activity one.

Objective Type	Objective
Primary	Describe factors influencing infant feeding decisions among WLHIV in the U.S., incorporating perspectives of women, influential individuals, and health care providers
	Estimate the prevalence of breastfeeding among women with HIV at select sites in the U.S.
Secondary	Describe possible approaches to support health and optimize care around infant feeding for WLHIV and their infants
	Explore current practices, perceived burden, benefits, and resources needed to support infant feeding in WLHIV in the U.S.

U.S., United States; WLHIV, woman living with HIV.

#### 2.2.2 In-Depth interviews and healthcare professional surveys

CA1 will consist of in-depth interviews (IDIs) with key stakeholders, including: WLHIV who are either pregnant or postpartum with a birth after February 2023 (when changes to HHS guidelines were announced; *n* = 50); influential individuals identified by WLHIV such as partners or spouses, family members, and friends who contribute to infant feeding decision-making (*n* = 25); and healthcare providers (HCP) and ancillary healthcare professionals (AHP) who care for WLHIV and/or their infants and participate in infant feeding decisions (*n* = 45) (Table 2). Each of these three stakeholder groups are mutually exclusive. A single IDI will be performed with each WLHIV and influential individual. A subset of HCP and AHP (approximately 24) will participate in a second IDI approximately 2 years after their initial interview, which will focus on their experiences in the context of the new policies of shared decision making around infant feeding and to assess thematic changes over time. These interviews are designed to be conducted at the end of the longitudinal cohort enrolled in CA2 to capture reflections on practice.

**Table 2:** Participants who will complete an in-depth interview.

Interview Group	Eligibility Criteria	Subgroups
Pregnant and Postpartum Women with HIV (*n* = 50)	WLHIV who are currently pregnant or gave birth after February 2023	Pregnant and considering breastfeeding (*n* = 10)
		Pregnant and not considering breastfeeding (*n* = 10)
		Postpartum and did not breastfeed for any duration (*n* = 10)
		Postpartum and breastfed for less than 4 weeks and have weaned (*n* = 10)
		Postpartum and breastfed for 4 weeks or longer (*n* = 10)
Influential Individuals (*n* = 25)	Spouses, partners, family members, or friends who are identified by the WLHIV participating in CA1 as being aware of her HIV status and contributing to infant feeding decisions	Individuals identified by pregnant or postpartum women who intend to or did breastfeed (*n* = 10-15)
		Individuals identified by pregnant or postpartum women who do not intend to or did not breastfeed (*n* = 10−15)
Healthcare Professionals (*n* = 45)	Healthcare provider or ancillary healthcare professional who participated in the care of at least five pregnant or postpartum WLHIV and/or their infants over the three years prior to entry	Providers with experience caring for pregnant/postpartum women with HIV (e.g., midwife, obstetrician, nurse, adult infectious disease specialist, advance practice provider; *n* = 15)
		Providers with experience caring for infants exposed to HIV (e.g., pediatrician, pediatric infectious disease specialist, nurse, advance practice provider; *n* = 15)
		Ancillary healthcare professionals with experience caring for pregnant and postpartum women living with HIV and/or their infants (e.g., social worker, lactation support provider, patient navigator, pharmacist, or doula; *n* = 15)

CA1, Core Activity 1; WLHIV, woman living with HIV.

All enrolled HCPs and AHPs who complete an interview will also complete a self-administered survey to collect information on individual knowledge, experience managing breastfeeding among WLHIV, current practices, and comfort with counseling on and management of infant feeding. Questions on the healthcare professional surveys were either adapted from prior publications or developed specifically for UPLIFT (73).

IDIs will be conducted using semi-structured interview guides. The interviews will explore the multifaceted process of infant feeding decision-making to establish important themes and patterns. Four interview guides were created by the study team for each participant group: pregnant WLHIV; postpartum WLHIV; influential individuals; HCPs and AHPs. The guides were reviewed and refined by the protocol team, including community team members with lived experience, HCPs, and breastfeeding and HIV research experts. The interview guides were specifically designed to elicit participant-informed suggested approaches to improving support around infant feeding for WLHIV and their infants.

The interview team is comprised of three senior qualitative researchers (KH, EAB and IVA) and six junior qualitative researchers. Strong effort was made to ensure a diverse composition of the team including race, ethnicity, language, location of birth, and other demographic characteristics. The team participated in five hours of study-specific training on conducting interviews, data collection and management, rapid thematic analysis, and safety procedures. All team members also participated in a series of mock interviews, analysis, and team review and feedback.

IDIs will be conducted virtually using Zoom. At the start of each IDI, a site staff member will join the Zoom meeting to confirm the correct identity of the participant and their PID. IDIs can be conducted with interpreters to allow enrollment of a diverse population of WLHIV. Each interview will be audio recorded, transcribed, and analyzed using rapid thematic analysis (74).

#### 2.2.3 Core Activity 1 study population

To comprehensively examine infant feeding decision making and experiences in the context of HIV in the U.S., it is essential to include the perspectives of multiple participant groups who influence, support, and enact these decisions (Table 2). Approximately 50 WLHIV will participate in a single IDI. These 50 WLHIV will be recruited from 5 sub-groups with approximately 10 individuals in each, including: (1) pregnant women who are considering breastfeeding; (2) pregnant women who are not considering breastfeeding; (3) postpartum women who did not breastfeed for any duration; (4) postpartum women who breastfed for less than 4 weeks and have weaned; (5) postpartum women who breastfed for 4 weeks or longer.

WLHIV participants will each be asked to identify one individual (e.g., partner, family member, or other) who both: 1) is aware of their HIV status; and 2) played an important role in influencing their infant feeding decision (“influential individuals”). It is anticipated that not all WLHIV will be able to identify an influential individual who meets these criteria. Approximately 25 influential individuals will be selected to complete a single IDI. Approximately half of the enrolled influential individuals will be identified by WLHIV who, at the time of enrollment, have breastfed (if postpartum) or intend to breastfeed (if pregnant) and half by WLHIV who do not intend to or did not breastfeed.

Approximately 45 HCPs or AHPs with experience caring for WLHIV and/or their infants will be interviewed. These 45 participants will be recruited from 3 sub-groups of approximately 15 each, including: (1) HCP with experience caring for WLHIV (e.g., midwife, obstetrician, nurse, adult infectious disease specialist, advance practice provider); (2) HCPs with experience caring for infants of WLHIV (e.g., pediatrician, pediatric infectious disease specialist, nurse, advance practice provider); (3) AHPs who are involved in the care of pregnant and postpartum WLHIV and/or their infants (e.g., social worker, lactation support provider, patient navigator, pharmacist, or doula).

The sample sizes for the IDI groups and sub-groups were selected to achieve a maximum variation sample inclusive of the wide geographic and demographic populations of interest and to meet standard qualitative requirements for thematic saturation (i.e., pattern explanation) within groups, while also providing the opportunity for thematic saturation within specific sub-groups and potentially across sub-groups (75–77). While data saturation is not required for the qualitative analysis approach, the planned minimum sample size per stratified group (*n* = 10) fits within the range recommended for descriptive qualitative analyses in homogenous groups (78, 79). Enrollment will be assessed weekly to ensure a sufficiently diverse sample of IDI groups and sub-groups and modifications will be made to ensure diversity and variability. Enrollment will be continuous and close when the target sample is reached.

#### 2.2.4 Data collection for IDIs and healthcare professional surveys

All IDIs will be audio-recorded through Zoom with a backup recording using Voice Notes. The audio files will be automatically stored on a secure server at the University of Colorado and subsequently transcribed by an approved AI software (Avidnote), which has strict privacy and data access policies. Transcripts will be stored on the same secure server at the University of Colorado and deleted within Avidnote after transcription. A member of the qualitative team will review each transcript alongside the audio to ensure accuracy and make any needed revisions. Names or other identifying information divulged during the interview will be redacted from the transcripts. Finalized transcripts will be linked to the participant only by their PID. The audio files and transcripts will be password protected. After analysis is complete, de-identified data files (e.g., transcripts and coding frameworks) from qualitative interviews will be stored at Frontier Science's File Exchange Utility (FEU), a secure web-based facility and then processed into the IMPAACT Central Database for long term storage. The audio recording will be deleted (from all servers) after the transcription has been certified as accurate and coding has been completed.

Healthcare professionals will complete self-administered electronic surveys directly in Medidata Patient Cloud using tablets, or in fillable pdf files with the data subsequently entered into Medidata RAVE by site staff.

#### 2.2.5 Qualitative analysis

Qualitative data from IDI transcripts will be analyzed using a combined deductive and inductive rapid thematic analysis approach to more efficiently disseminate information to guide decision-making around infant feeding in the context of HIV. Rapid thematic analysis enables researchers to identify relevant themes efficiently and systematically without the long timelines of traditional line-by-line qualitative coding methods. Our rapid thematic analysis approach will use a team to extract insights and key points from the IDIs into matrices to provide easy interpretation of the meanings in the data (80, 81). For each population group (i.e., pregnant women, postpartum women, influential individuals, and HCPs/AHPs), we will create a templated summary table with pre-specified domains (*a priori* deductive codes) based on the interview guides, existing literature, and study objectives, with a column to input key points (inductive) and illustrative quotes for each interview transcript. The table will have the option to input additional inductive domains that may emerge from the IDI transcripts as well. For key points, the team will use short understandable bullets with one data point per bullet to paraphrase and summarize, which are not intended to be interpretative. Verbatim quotes from the interview transcript will be copied to illustrate key points where appropriate and confirmability of data interpretation.

After completion of the summary tables for each transcript, we will consolidate all data by participant type into matrices. Each matrix will have the domains listed as columns, and all transcripts listed in rows. The key points and illustrative quotes from each transcript will be included in each row in the relevant domain. This enables quick review to identify reoccurring and discrepant themes within each domain. The most salient themes and illustrative quotes will then be summarized with affinity maps and other tools during qualitative team meetings, including team members from The Well Project with lived experience, and stakeholder workshops used to establish key findings and conclusions.

To establish calibration, all analysts will complete a summary table for the same single transcript within each group and meet to compare, discuss, and refine. If needed, an additional calibration process and meeting will occur. Once 50% of summary tables are complete for each IDI group, the qualitative team will also hold a calibration check-in meeting to review reoccurring key points and emergent domains, and the potential need to re-calibrate if large discrepancies are observed among the analysts. If re-calibration were to occur, previously coded transcripts would be re-coded in summary tables and reviewed by the team until calibration is achieved.

#### 2.2.6 Quantitative analysis

Quantitative data collected from healthcare professional surveys will be analyzed descriptively. Frequencies and percentages for categorical data, and mean, standard deviation, median, Q1 and Q3 for continuous data will be reported as a whole, by site, and by provider type. The individual provider survey responses will be linked to the provider transcripts during final analysis and used to help contextualize themes and interpretations.

#### 2.2.7 Landscape analysis

Each participating site will complete site-level surveys in REDCap hosted by Frontier Science. Monthly surveys will collect the total number of WLHIV who deliver and who initiate breastfeeding at each site to estimate site-level prevalence of breastfeeding among WLHIV. We will use baseline and annual site surveys to collect data on site characteristics, current approaches to infant feeding counseling and the clinical management of WLHIV and their infants, and site-determined strengths and challenges in the care of breastfeeding WLHIV. No individual-level data, such as HIV-related laboratory results, will be collected in the landscape surveys. Approaches used to support health and optimize care around infant feeding for WLHIV and their infants will be gathered and compared across sites. Quantitative data collected from site-level surveys will be analyzed descriptively by reporting frequencies of various descriptive measures by site, staffing model, etc. Survey data will also be used to calculate the proportion of postpartum women with HIV within each site and aggregated across sites who breastfeed for any duration, and to create a 95% confidence interval to estimate the proportion of WLHIV who choose to breastfeed. These data will be used to construct a landscape analysis that will provide a broad overview of the trends, emerging issues, and opportunities in supporting infant feeding decision making for U.S. WLHIV. Findings from the repeat site-level surveys will help assess changes in the field as a whole and at the site level across the years of the study.

### 2.3 Core Activity 2 (CA2)

#### 2.3.1 Core Activity 2 objectives

The primary, secondary, and exploratory objectives of CA2 are presented in Table 3.

**Table 3:** Objectives of Core Activity two.

Objective Type	Objective
Primary	Describe infant feeding practices and identify aspects of clinical, behavioral, and social factors associated with different infant feeding choices and durations among WLHIV
	Identify factors associated with detectable HIV RNA or DNA in breast milk
Secondary	Describe key outcomes among WLHIV according to infant feeding choice
	Describe key outcomes among infants of WLHIV according to infant feeding choice
	Determine the costs and cost-effectiveness of different infant feeding choices
	Identify factors associated with detectable HIV RNA in the plasma of WLHIV who breastfeed
	Describe the temporal relationships between detectable HIV DNA or RNA in breast milk and detectable plasma HIV RNA among WLHIV
Exploratory	Explore risk factors for infant HIV acquisition during breastfeeding

WLHIV, woman living with HIV.

#### 2.3.2 Longitudinal cohort

**Figure 3:**
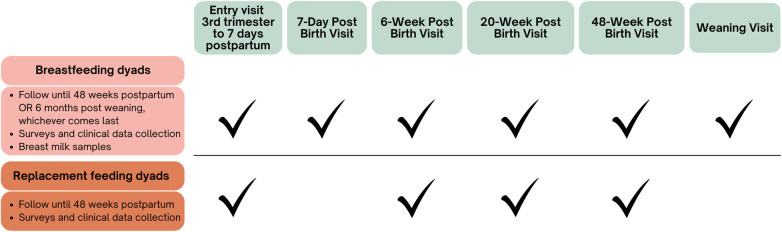
Study visits in Core Activity Two. Checkmarks indicate the timing of required study visits for breastfeeding and replacement feeding mother/infant dyads.

Core Activity 2 will include a prospective observational longitudinal cohort of WLHIV and their liveborn infants (*n* = 450). Approximately half of WLHIV in CA2 will be WLHIV who intend to breastfeed or are breastfeeding at enrollment. Study visits will be conducted at entry and 6 (+/−2), 20 (+/−4), and 48 (+/−4) weeks post-birth for all participants. Breastfeeding WLHIV-infant dyads will complete additional study visits at 7 (+/−7) days post birth, at the time of any breastfeeding complications, and at weaning (Figure 3). Complication visits will be triggered by a plasma viral load of >50 copies/mL for the WLHIV and/or a breast problem, including painful breast swelling, red or shiny skin, cracked or bleeding nipples, blocked ducts, abscess, rash or dermatitis, with or without associated fever. Participants will be instructed to notify site staff immediately when symptoms of a breast problem occur so that a complication visit can be scheduled in a timely fashion. All participants will be followed until infant HIV testing is complete and at least 48 weeks post-birth. Participants who have completed infant HIV testing by 48 weeks post-birth will exit the study at 48 weeks. Breastfeeding participants who have not completed infant HIV testing by 48 weeks post-birth will exit the study at 24 weeks after the infant's last exposure to breast milk, or at 96 weeks post-birth, whichever comes first.

#### 2.3.3 Core Activity 2 study population

The longitudinal cohort will enroll approximately 450 women with HIV who are ≥28 weeks pregnant or up to 7 days postpartum and their live-born, singleton infants. A cut off of at least 28 weeks’ gestation was selected to reduce enrollment of women who may experience a pregnancy loss and based on literature demonstrating that infant feeding decisions may change early in pregnancy (82).

#### 2.3.4 Core Activity 2 data collection

At each study visit, data will be collected, including maternal and infant medical and medication history, infant feeding history, and participant anthropometrics (height and weight for WLHIV; weight, length and head circumference for infants). Additional clinical data and laboratory results will be abstracted from the medical record. Trained research staff at UPLIFT sites will collect clinical and laboratory data and enter it into Medidata Rave.

Self-administered electronic surveys will be completed by WLHIV at each study visit, collecting data on demographic characteristics, socioeconomic status, physical and mental health, and infant feeding intentions and experiences. Survey instruments were adapted from those used in other studies [e.g., the Health Outcomes around Pregnancy and Exposure to HIV/Antiretrovirals (HOPE) study]; sourced from previously published surveys; or developed specifically for UPLIFT (Supplementary Table) (83). Participants will enter survey data directly into Medidata Patient Cloud using tablets during study visits. Research staff will be available to assist as needed. Surveys will be available in multiple languages, either using existing validated non-English versions or via certified translation, and/or can be completed with an interpreter to facilitate enrollment of a diverse cohort of WLHIV.

An IDI will be conducted at the 48-week post-birth visit with approximately 20 postpartum participants who breastfed and approximately 20 participants who never breastfed. These interviews will focus on evolving infant feeding experiences, decision-making over time, and perceived supports and challenges.

#### 2.3.5 Breast milk specimen collection

Breast milk samples will be collected at each study visit from breastfeeding participants until weaning is complete. Milk will be expressed from both breasts according to participants’ preferred method, which may include hand-expression or the use of a manual or electric breast pump. A minimum of 15 mL and maximum of 60 mL of breast milk will be collected at most visits, with smaller minimum and maximum volumes allowable in the first 14 days postpartum, when the milk supply is not yet established. Milk will be refrigerated immediately and processed within 6 h of collection. Milk will be centrifuged and the fat layer discarded. The aqueous layer of milk will be aliquoted and cryopreserved. Breast milk cells will be cryopreserved as a nonviable cell pellet. Breast milk samples will be shipped to a centralized laboratory for batched analysis. HIV RNA will be measured in the aqueous milk compartment and HIV DNA will be measured in the breast milk cells using quantitative PCR. Results of breast milk HIV testing will not be shared with sites nor participants, because the assays will not be performed in real time, and the research assays are not approved for clinical use.

#### 2.3.6 Case review of breastfeeding-associated infant HIV transmission

Infant HIV acquisition is expected to occur very infrequently, if at all, in the UPLIFT study. In the event that an HIV transmission to a breastfeeding infant is identified during the study, an adaptation of the Fetal & Infant Mortality Review-HIV Prevention Methodology (FIMR-HIV) will be performed (84, 85). The FIMR-HIV process will include a structured maternal interview, collation of medical and medication history for the mother and infant and maternal self-administered survey data collected at study visits (Supplementary Table), and a virtual collaborative case review. Case reviews may include site staff, clinical providers for the mother and infant, select study team members (as applicable), and other relevant individuals such as representatives from the local public health department or patient advocates. Site staff will complete a standardized case review form documenting participant characteristics, a deidentified case summary, proposed recommendations, and an action plan. The goal of FIMR-HIV is to identify and address missed opportunities for prevention of HIV transmission.

#### 2.3.7 Core Activity 2 quantitative analysis

In order to describe infant feeding practices and identify aspects of clinical, behavioral, and social factors associated with different infant feeding choices and durations among WLHIV (CA2 co-primary objective), we will describe infant feeding among CA2 participants in several ways. Participants will be categorized by infant feeding method: breastfeeding (defined as receiving breast milk from the mother with HIV for any duration) and replacement feeding (defined as no exposure to breast milk from the mother with HIV). We will report the number and proportion of women who initiate breastfeeding among those who intended to breastfeed, and those who complete their intended duration of breastfeeding.

Several baseline factors will be evaluated as predictor variables to explore factors associated with type of infant feeding. These baseline factors include: geographic location (site); year enrolled; demographic factors (race, ethnicity, age, internationally born, language, marital status); clinical factors (gravity/parity, timing of HIV diagnosis, viral load, CD4 count, ART adherence); social and structural determinants of health (education, employment, income, housing, food insecurity, transportation); healthcare access; health literacy; HIV disclosure; HIV-related stigma; interpersonal violence; substance use; and depression/anxiety symptoms. Three different logistic regression models will be used to identify the statistically significant baseline factors that are associated with the binary outcomes: breastfeeding vs. replacement feeding, exclusive breastfeeding vs. exclusive replacement feeding, and whether a participant completes their intended duration of breastfeeding. Additionally, a generalized linear regression model will be used to determine the baseline factors associated with the continuous outcome, duration of breastfeeding. No adjustments for multiple comparisons are planned. For each model, the marginal regression for each factor will be applied to screen for significant factors first, and then the best subset regression variable selection approach will be used to determine the significant factors in the regression model. For linear regression models, standard diagnostic procedures will be conducted to evaluate key model assumptions, including linearity of covariate effects, normality of residuals, homoscedasticity, independence of observations, and the absence of influential outliers. Linearity will be assessed using various plots and summary diagnostics, and homoscedasticity through residual scatterplots and formal tests when appropriate. For logistic regression models, assumptions will include correct specification of the logit link, linearity of continuous covariates on the log-odds scale, absence of multicollinearity, and lack of overly influential observations; these will be assessed using diagnostic plots, variance inflation factors, and influence measures such as delta-beta statistics. If assumption violations are identified, prespecified corrective actions will be implemented.

Missingness of data will be summarized by groups and key covariates, and graphical and tabular diagnostics will be used to assess whether data are plausibly missing completely at random (MCAR), missing at random (MAR), or missing not at random (MNAR). For the primary analyses, regression models will be fit using methods appropriate to the assumed missingness mechanism, with complete-case analysis applied only when missingness is minimal and unlikely to introduce bias. When missing data are non-negligible, multiple imputation using fully conditional specification will be employed.

We will describe the proportion of milk samples among breastfeeding participants that contain detectable HIV RNA and DNA (co-primary objective). Also, we will use mixed effects logistic regression models to identify statistically significant factors associated with detectable HIV in milk. These will include baseline and other factors including time of sample collection (i.e., study visit); presence of mastitis; presence of nipple trauma; presence of mixed feeding; and presence of plasma viremia in plasma sample collected per standard of care nearest (and within +/- 14 days) to the date of milk sample collection.

We will use logistic and generalized linear regression models to determine whether infant feeding method is associated with maternal mental health and quality of life survey scores, decision regret, satisfaction with infant feeding choice, and infant growth and development, and to identify factors associated with detectable plasma viral load. The temporal relationship between detectable HIV in breast milk and in plasma will also be described (secondary objectives).

Transmission of HIV to infants via breastfeeding is unlikely to occur in this study given the low risk of transmission among WLHIV on ART with sustained viral suppression, the close monitoring and regular follow up of participants in a research study, and the sample size of anticipated breastfeeding WLHIV. We will describe any infant HIV transmissions, including the maternal plasma viral load and presence of detectable HIV in milk, along with additional data gathered from FIMR-HIV review (exploratory objective).

#### 2.3.8 Power calculation

Sample size was determined using the primary objective of CA2 to determine which baseline factors are significantly associated with the decision to breastfeed among postpartum WLHIV. Assuming that the proportion of WLHIV who breastfeed is 150 among 450 recruited participants and a 5% loss to follow-up rate, we calculated that the minimum detectable odds ratio (MDOR) for detecting an association between breastfeeding and continuous exploratory variables would be 1.34 for each 1 standard deviation increase (with 80% power and a significance level of 0.05 for a two-sided test). For a binary explanatory variable with a Binomial distribution where π = 0.5, the MDOR is 1.75 (86, 87). Since the analysis uses logistic regression to evaluate associations between baseline factors and a binary outcome, the odds ratio is the natural measure of effect size. The true effect sizes of exploratory predictors are unknown *a priori*, so the MDOR provides a transparent way to quantify the smallest association that can be detected with 80% power at a two-sided *α* = 0.05, given the anticipated event rate, sample size, and predictor distribution. Thus, MDOR reflects the statistical sensitivity of the study and aligns directly with the planned modeling framework.

The co-primary objective of CA2 is to determine whether any factors are associated with the presence of detectable HIV RNA or DNA in breast milk. Because breastfeeding transmission is expected to occur rarely, if at all, in the UPLIFT study, the detection of HIV in breast milk was chosen as a proxy for the risk of transmission. Mastitis is a common complication during breastfeeding that has been associated with an increased risk of detectable HIV RNA or DNA in milk, and with HIV transmission (88–92). Mastitis was used as an example of a factor that may increase the presence of detectable HIV RNA or DNA in milk in a power calculation. Data are not available to determine the prevalence of detectable HIV RNA in milk among lactating women with HIV on integrase inhibitor-based ART; however, several studies of lactating women with HIV on nevirapine-based ART regimens demonstrated a prevalence of HIV RNA in milk of 10%–30% (93–95). We assumed that the prevalence of HIV RNA in milk would be lower among lactating women receiving modern potent ART (e.g., ∼5%). The power calculation includes the following assumptions: 1) Among participants treated with potent modern ART, up to 5% may have detectable HIV RNA in milk; 2) 10%–15% of participants who breastfeed will experience mastitis at some point during lactation; 3) in the pre-ART era, mastitis resulted in a 10-fold increase in the likelihood of detectable HIV RNA in milk (88, 89, 96, 97). If we suppose that 10%–15% of 150 participants experience mastitis and the rates of detectable HIV RNA in milk of those with and without mastitis are 50% and 5% respectively, then with a significance level of 0.05, we have 98%–99% power to detect a difference in the proportion of milk samples that contain HIV RNA between participants with and without mastitis with a sample size of 150 breastfeeding participants.

#### 2.3.9 Qualitative and mixed methods analysis

IDIs conducted in CA2 build on themes identified in CA1 and are analyzed using the same rapid thematic analysis approach. Across CA1 and 2, qualitative data from IDIs are summarized using matrix-based methods to identify key themes, patterns, and changes over time related to infant feeding decision-making, experiences, and care needs among WLHIV and other influential individuals.

**Figure 4:**
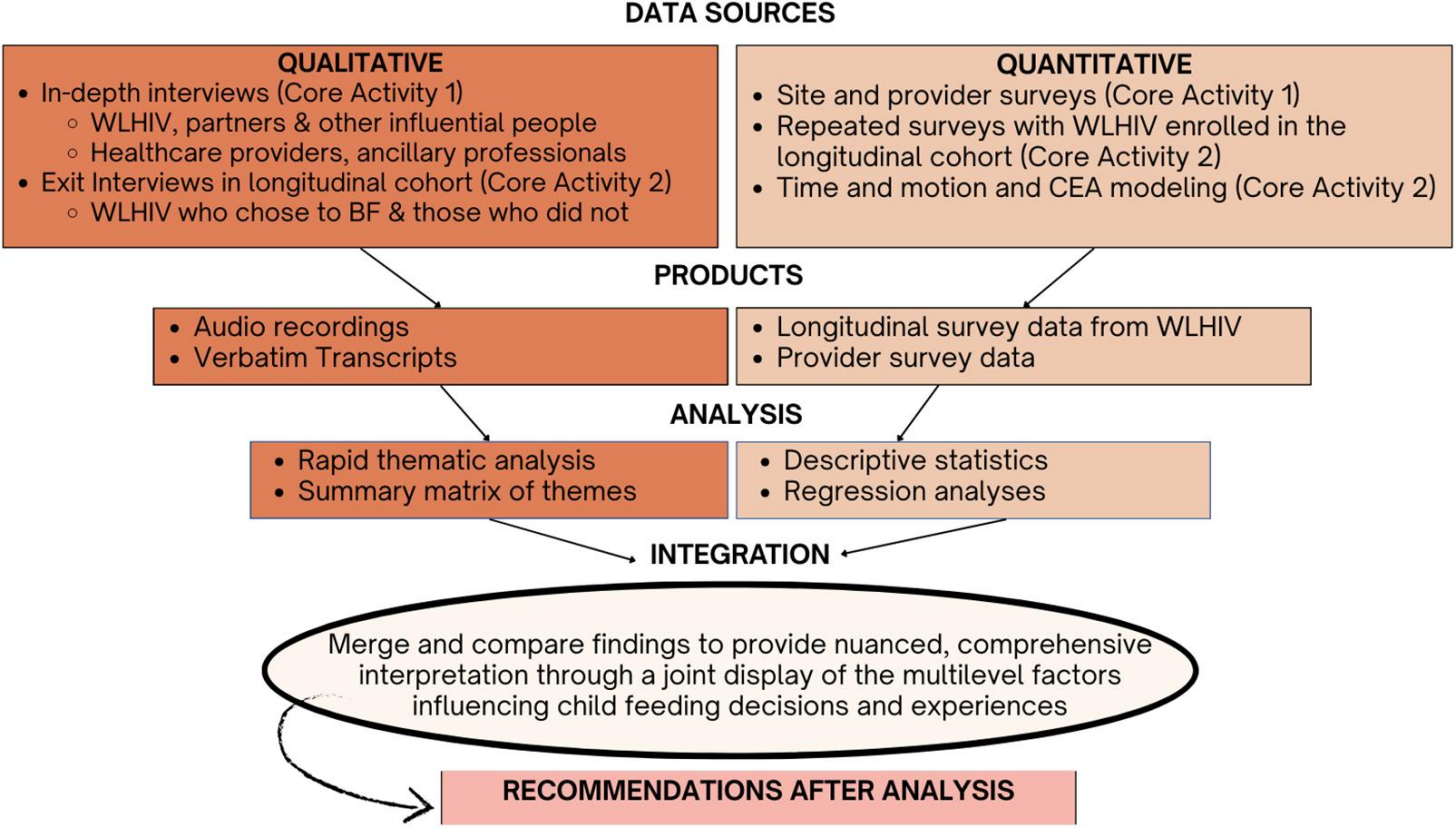
Convergent mixed-methods analysis approach for UPLIFT. Qualitative and quantitative data will be analyzed separately and then integrated in a convergent mixed-methods analysis. BF, breastfeed; CEA, Cost Effectiveness Analysis; WLHIV, women living with HIV.

UPLIFT uses a convergent mixed-methods analytic approach in which qualitative and quantitative data are analyzed separately and then integrated (Figure 4). We will integrate qualitative and quantitative findings through a joint display table, which will align themes from in-depth interviews (CA1) with corresponding quantitative measures and trends (CA1 and CA2). This joint display is used to identify areas of convergence, divergence, and expansion, allowing comparison of lived experiences and perspectives with observed quantitative patterns over time. This mixed-methods integration supports a comprehensive and contextualized understanding of infant feeding decisions, practices, and care needs among pregnant and postpartum WLHIV in the U.S.

#### 2.3.10 Cost and cost-effectiveness analyses

An economic evaluation will be conducted to estimate the cost and cost-effectiveness of infant feeding strategies for WLHIV following the recommendations of the Second U.S. Panel for Cost-effectiveness in Health and Medicine (98). We will employ a societal perspective and use a micro-costing approach to estimate provider costs for each feeding strategy. In addition, we will incorporate participant costs and potential productivity losses from labor market participation or household production as components of overall cost. This analysis is descriptive in nature and is intended to characterize the range and distribution of costs associated with each infant feeding strategy rather than to evaluate differences by employment status or workplace factors. We will calculate mean total costs for each mother/infant dyad for breastfeeding and replacement feeding and examine variation in cost components by infant feeding strategy. Cost data will be collected through self-administered participant surveys (Supplementary Table) and time and motion studies to estimate labor and participant time costs. Labor costs will be calculated using labor time (hours spent on infant feeding activities) and national wage rates and fringe benefits by job classification. Participant survey data will be used to characterize patient costs including expenses for infant feeding as well as time spent on infant feeding activities and associated losses in productivity. All CA2 participants will complete cost surveys at 6, 20, and 48 weeks. The time and motion evaluations will be conducted at a subset of sites, selected based on accrual projections and services offered. Cost data elements that will be used to estimate and compare the costs associated with breastfeeding and replacement feeding for mother/infant pairs are detailed in the cost inventory (Table 4).

**Table 4:** Proposed cost inventory by infant feeding strategy.

Cost Category	Breastfeeding	Replacement Feeding
Program Delivery Costs	Breastfeeding support and monitoring (e.g., lactation support, counseling follow-up visits medication)	Infant feeding support and monitoring (counseling, follow up visits, medication)
Materials and Supplies	Consumables, educational materials	Consumables Educational materials
Program Administrative and Costs	Labor costs for supervision, overhead	Labor costs for supervision, overhead
Patient Direct Costs	Out-of-pocket feeding costs, breast pumps & accessories, bottles, storage supplies	Out-of-pocket feeding costs, formula, bottles, sterilization supplies
Patient Time Costs and Productivity Losses	Patient time spent breast feeding and attending feeding-related medical visits	Patient time spent replacement feeding and attending feeding-related medical visits
Averted Health Care Costs	Averted medical care costs attributable to breast feeding	Averted medical care costs attributable to formula feeding

We will use decision analytic modeling using a Markov model to extend the analysis and estimate the incremental cost per quality-adjusted life year (QALY) of breastfeeding compared with replacement feeding (99, 100). The model will incorporate infant and maternal morbidity and mortality, as well as HIV transmission and averted illness by infant feeding strategy. It will be parameterized with UPLIFT study data and data from the published literature including the 2025 Agency for Healthcare Research and Quality (AHRQ) systematic review of breastfeeding-related health outcomes in infants and children (101). We will explore uncertainty in the incremental cost-effectiveness ratio (ICER) with sensitivity analyses accounting for plausible variation in applicable cost and outcome parameters.

### 2.4 Core Activity 3 (CA3)

#### 2.4.1 Core Activity 3 objectives

The objective of CA3 is to describe the usability, acceptability, appropriateness, and feasibility of a registry for postpartum WLHIV who breastfeed in the U.S.

#### 2.4.2 Registry development

Within UPLIFT, a registry will be piloted and iteratively developed. The prototype registry will be developed with input from protocol team members with expertise in health registries, care of WLHIV and their infants, and community members with lived experience. The registry will be informed and refined by qualitative interviews and human-centered design (HCD) workshops with WLHIV and HCPs, incorporating feedback from pilot testers. Data collected from WLHIV participating in CA1 interviews will assess the acceptability of a registry and solicit input on recruitment, data collection, and data usage. Additional CA1 interviews with HCPs will obtain perspectives on data collection, workload, feasibility, and acceptability. Virtual HCD workshops with WLHIV (two workshops, *N* = 20), HCP (two workshops, *N* = 20), and community advocates (one workshop, *N* = 8) will be held to co-refine the registry prototype, including consideration of appropriate and non-stigmatizing terminology, ongoing engagement of WLHIV and HCPs, and future scale-up. Although the term “registry” is used throughout this protocol for clarity, the final name and framing of the registry will be informed by participant input and refined through the HCD process.

#### 2.4.3 Registry piloting

The registry will be piloted at all 12 UPLIFT sites. Consent will be obtained from participants during the pilot phase for the inclusion of de-identified data in the voluntary registry. Breastfeeding WLHIV will be enrolled within 7 days after delivery and prospectively followed through weaning and completion of infant HIV testing.

#### 2.4.4 Core Activity 3 study population

The pilot registry will include data from a minimum of 50 study-naïve WLHIV-infant dyads who enroll in CA3, with each of the 12 sites entering data on at least 3 mother/infant pairs. WLHIV who are enrolled in the longitudinal cohort of CA2 will also be eligible to co-enroll in CA3, for a total of up to 200 WLHIV-infant dyads included in the pilot registry.

Approximately 30 pilot registry testers who enter registry data or supervise data entry will participate.

#### 2.4.5 Data collection

Site staff (pilot registry testers) will abstract demographic, clinical and laboratory data from the medical records of WLHIV and their infants and enter the data into a REDCap database hosted by Frontier Science (69). Sites will complete data entry at 2 time periods: 1) after delivery of the infant; and 2) after the infant has weaned from breast milk and completed all HIV testing. Recognizing the sensitivity around data collection and monitoring in WLHIV, the registry will employ robust data security measures. No personally identifiable information will be recorded in the registry, and only the participant's affiliated clinical site will maintain a link between the participant and their anonymized code.

To inform iterative revisions of the registry, approximately 30 pilot testers entering data or overseeing data entry will complete standardized surveys on its usability, acceptability [Acceptability of Intervention Measure (AIM)], feasibility [Feasibility of Intervention Measure (FIM)], and appropriateness [Appropriateness of Intervention Measures (IAM)] after entering data on at least three mother/infant dyads (102). Additionally, pilot testers will provide feedback after entering data on each mother/infant dyad to describe their overall experience, ease of use, time required, and suggestions to improve the registry.

#### 2.4.6 Core Activity 3 analysis

The CA3 analysis will include a review of the completeness of the data (proportion of entries with 100% data completion for mandatory fields and 85% completion for optional fields) and investigate commonly missed data points to understand the barriers in collecting these data. The types of providers that are most likely to enter data in the registry will be summarized, the aggregated demographic and clinical data of the entered participants will be reported, and any unanticipated safety events will be described. For implementation outcomes, the median score, from 0 (worst) to 5 (best), on the usability, AIM, FIM, and IAM surveys will be reported. Given the pilot nature of the registry, no formal hypothesis-driven analysis of the data entered will be performed.

#### 2.4.7 Scale-Up of registry

Insights from the pilot study will be used to develop the National Observational Reporting on Infant feeding to Support women with HIV (NOURISH) Lactation Registry. NOURISH will engage additional sites throughout the U.S. to prospectively contribute voluntary data to the registry. Insights from the pilot study will be used to develop a detailed protocol, implementation plan, governance structure, and framework for accountability and oversight of the NOURISH registry as it is scaled up to sites outside of the IMPAACT network. The long-term goals of the NOURISH registry are to: (a) collect geographically diverse and representative data on WLHIV across the U.S. who breastfeed and their infants; (b) observe safety and health outcomes of infants of WLHIV who breastfeed and identify any unanticipated risks; (c) address knowledge gaps around infant feeding to inform the care of WLHIV and their infants; and (d) provide a rich source of data to generate hypotheses for future research.

## 3 Discussion

The 2023 change to infant feeding guidelines for WLHIV in the U.S., moving to shared decision making and support for breastfeeding in WLHIV who are virally suppressed on ART, created the need for rigorous research to address remaining knowledge and implementation gaps. Lack of data from high-income settings, such as the U.S., continues to fuel uncertainty regarding breastfeeding among WLHIV and for their HCP. Findings from the UPLIFT study will address these gaps and inform practice and policy regarding infant feeding for WLHIV in the U.S. By utilizing a multi-site mixed-methods approach, integrating qualitative perspectives of WLHIV and their care teams with longitudinal prospective data, as well as piloting a database for ongoing understanding of infant feeding trends and outcomes in WLHIV, the UPLIFT study is poised to influence infant feeding counseling, clinical management, health system implementation, and future research for WLHIV who breastfeed in the U.S. and beyond.

CA1 will collect comprehensive insights from WLHIV and other key stakeholders regarding their perspectives and experiences with infant feeding and will include a landscape analysis to characterize existing clinical practices, resources, and contextual factors shaping infant feeding counseling and support. Interviews will intentionally include a diverse group of participants, including WLHIV who have and have not breastfed while living with HIV since the guidelines change, as well as pregnant WLHIV who are currently making infant feeding decisions. Centering the perspectives and experiences of WLHIV is critical to understanding infant feeding decision-making and to informing clinical practice and policy that is responsive to real-world priorities, lived experience, scientific evidence, and individual values (31, 103). Critically, CA1 will include influential individuals who contribute to infant feeding decisions. While autonomy is a priority for WLHIV, existing research demonstrates the importance of partners, spouses, family, and others in influencing infant feeding decisions (104, 105). Furthermore, lack of disclosure of HIV status is often a barrier to the inclusion of influential individuals in perinatal HIV services (106). Including the perspectives of these key individuals will provide important interpersonal and social context and may inform strategies to enhance engagement in future infant feeding counseling approaches. Finally, HCPs and AHPs comprise the care team for WLHIV and their infants and are directly influencing and implementing infant feeding guidance and management. Recent literature shows significant variability between HCPs regarding comfort and practices around infant feeding for WLHIV (61–63). Thus, including HCP and AHP in interviews is essential to understanding provider perspectives. In sum, we aim to provide the most comprehensive description to date of attitudes, experiences, and clinical practices related to infant feeding for WLHIV in the U.S.

In CA2, we will enroll a large prospective observational cohort of WLHIV in the U.S. who do and do not choose to breastfeed. This will be the first cohort study of its size to enroll in a high-income setting, where breastfeeding WLHIV have access to frequent virologic testing, maternal ART, infant antiretroviral prophylaxis, and replacement infant foods. This cohort provides the opportunity to examine the impact of scenarios traditionally considered to increase the risk of breastfeeding transmission (e.g., mastitis, mixed feeding) on the presence of HIV in milk among women who are largely virologically suppressed on ART. Closing these critical knowledge gaps will provide the evidence needed to inform guideline development and strengthen future management strategies for WLHIV facing breastfeeding complications in high-income settings. The longitudinal interviews in CA2 will complement cohort data by gathering data on evolving infant feeding experiences, decision-making, and care needs over time. Finally, the cohort will generate a rich prospective dataset and comprehensive set of breast milk samples that can be explored in future sub-studies to answer numerous questions about infant feeding choices and practices, the factors that inform those choices, and WLHIV and infant outcomes.

The pilot registry in CA3 will establish the foundation for a sustainable data collection infrastructure that can provide valuable insights into the long-term safety and effectiveness of breastfeeding in WLHIV. This will be the first registry of its kind to aggregate data on breastfeeding outcomes from WLHIV and their infants across the U.S. The registry will be iteratively developed with input from community members with lived experience and providers across 12 U.S. sites, with a long-term goal of scaling up to a voluntary, nationwide registry operating outside of research settings. If successful, the registry can be rapidly scaled to address ongoing knowledge gaps, identify critical epidemiologic trends, and inform a future research agenda for infant feeding among WLHIV in the U.S.

Strengths of the UPLIFT study include the inclusion of multidisciplinary protocol team members who contribute expertise in HIV care, pediatrics, obstetrics, maternal-child health, infectious diseases, breastfeeding, epidemiology, nursing, midwifery, lactation support, behavioral health, qualitative research, biostatistics, health economics, registry science, laboratory science, public health, and lived experience. Community members with lived experience are involved at every level of the study, from design to implementation to analysis. Further, the study will leverage geographically diverse locations at 12 sites across the U.S., providing geographic, cultural, and racial/ethnic representation and enhancing generalizability of findings. Across Core Activities, qualitative and quantitative data will be integrated using a convergent mixed-methods approach, yielding richer insights and a more comprehensive understanding.

Limitations of UPLIFT include that all study sites represent primarily urban, academic research institutions with some experience supporting WLHIV who breastfeed. The perspectives and concerns of WLHIV and healthcare staff from smaller, rural U.S. clinics and non-academic institutions may not be well represented. In addition, while rapid thematic analysis supports timely, structured synthesis of qualitative data across a large, multi-site study, this approach may limit the depth of interpretive analysis achievable with more prolonged or iterative qualitative methods. To mitigate this, analyses will be conducted by a multidisciplinary team, informed by community contributors, and will incorporate data from multiple participant groups and time points. There is the potential for social desirability and recall biases given that the interview and survey data are self-reported. The cohort study of 450 WLHIV, half of whom are expected to breastfeed, may not be large enough to identify factors associated with breastfeeding transmission of HIV, given that infant HIV acquisition is expected to occur very rarely. Therefore, we have opted to use the detection of HIV in milk samples as a proxy for the risk of transmission. Additionally, the study is observational in nature as it would not be ethical to randomize WLHIV to breastfeed or formula feed. However, repeated clinical observations and long-term follow-up of mother-infant dyads that do and do not breastfeed will enable exploration of key knowledge gaps that are unlikely to be studied otherwise in as highly rigorous of an approach.

Overall, the UPLIFT study will generate critical insights to advance patient-centered care and support informed infant feeding decision-making and management among WLHIV and HCP as U.S. institutions adapt to evolving national guidelines.

## 4 Ethics and dissemination

Advarra, the single Institutional Review Board of record for all sites in the UPLIFT study, reviewed and approved UPLIFT protocol version 1.0 (version date 05 November 2025) and study-specific materials on 24 November 2025. All participants will provide written informed consent for themselves and/or their infants, if applicable. As part of the informed consent process for CA2, WLHIV will be asked whether they agree to storage and future research testing of milk specimens remaining after protocol-specified testing has been completed. This storage and future research testing is optional and may be declined with no impact on other aspects of study participation. As part of the informed consent process for CA3, WLHIV will be asked whether they agree for their pilot data to be included in the NOURISH Lactation Registry, once established. This national registry consent is optional and may be declined with no impact on other aspects of study participation. Any future research using data or samples from UPLIFT must be approved following standard CDC and IMPAACT data request procedures. Participants will not be personally identified in data shared for future research.

Study results will be presented at local, national, and international conferences, published in peer-reviewed journals, and disseminated through lay summaries. In partnership with The Well Project, findings will also be disseminated through community-facing channels, including the organization's website, blogs, podcasts, and social media platforms, to ensure broad and accessible reach to women living with HIV and their communities.

## Group Members of the UPLIFT Protocol Team Not in the Author List

Maggie Albano, Craig Borkowf, Kate Buchacz, Ashley Cason, Tessa Crume, Deborah Gelaude, Gena Grant, Rachael Jeffrey, Benjamin Johnston, Kevin Knowles, Rose Lagattuta, Sonia Lee, Dayana Leon, Madison O’Brien, Lauren O’Connor, Anna Powell, Elizabeth Rivas, Anaïs F. Stenson, and Rachel Ward.
