# Supplementary material for: Understanding practices of lactation and infant feeding together with women with HIV: the UPLIFT observational study protocol
Source: Front Reprod Health. 2026 Jul 8;8:1856899. doi: 10.3389/frph.2026.1856899 (PMC13388547; doi:10.3389/frph.2026.1856899)
Supplement: Supplementary file 1 [file Table1.pdf]

Supplementary Material

Supplementary Table. Surveys for Participants in Core Activity 2

Instrument Name	Domains	Entry	7 Day Post Birth Visit [a]	Weeks Postpartum			Complication Visit [a]	FIMR Visit [a]	Weaning Visit [a]
				6	20	48			
Infant Caregiver Information	• Relationship of caregivers to child	✓	✓	✓	✓	✓	✓	✓	✓
Household Information	• Housing, household members<br>• Income<br>• Occupation	✓							
Participant Information	• Age, race, ethnicity, birthplace, language<br>• Education<br>• Marital/relationship status, partner involvement	✓							
Follow up Socio-Demographics/ Economics	• Marital/ relationship status, partner involvement<br>• Housing, household members<br>• Income<br>• Occupation				✓			✓	
Pregnancy and Infant Feeding History	• Prior pregnancies and infant feeding decisions	✓							
Infant Feeding Knowledge, Preferences, and Intentions [b]	• Infant feeding plans and decision-making<br>• Sources of infant feeding information<br>• Infant feeding knowledge, beliefs<br>• Goal duration of breastfeeding	✓							
Delivery Experience [c]	• Support of delivery center for infant feeding decision		✓						
Ages and Stages [d]	• Child developmental screening tool			✓	✓	✓			
Infant Feeding Assessment [e]	• Types, amounts of foods baby is consuming<br>• Age at introduction of formula and/or solid foods<br>• Age at weaning, reasons for weaning	✓	✓	✓	✓	✓	✓	✓	✓

Decision Making [f]	• Decisional conflict re: infant feeding decision	✓							
Replacement Feeding Survey [g]	• Comfort with/ concerns about decision to replacement feed			✓	✓	✓			
Breastfeeding Issues Follow Up Survey [a]	• Breastfeeding complications, impact on feeding method						✓	✓	
Breastfeeding Self-Efficacy Scale [a, h]	• Confidence in one's ability to breastfeed		✓	✓	✓	✓			✓
Beginning Breastfeeding Survey – Cumulative [a, i]	• Perceptions of breastfeeding success		✓	✓	✓	✓			✓
Maternal Breastfeeding Evaluation Scale [a, j]	• Emotions and thoughts around breastfeeding		✓	✓	✓	✓			✓
Participant Cost Survey (Pre-Birth)	• Time spent in prenatal visits (including lactation specialists)<br>• Infant feeding out of pocket costs			✓					
Participant Cost Survey (Post-Birth)	• Time spent in postnatal visits (including lactation specialists)<br>• Time spent feeding infant<br>• Infant feeding out of pocket costs				✓	✓			
Decision Regret Scale [k]	• Reflection on infant feeding decision				✓	✓			✓
Infant Feeding Experience [l]	• Satisfaction, guilt, stigma re: infant feeding<br>• Disclosure concerns re: infant feeding<br>• Child Protective Services concerns re: infant feeding				✓	✓			✓
HIV Medications [m]	• Medication consistency	✓	✓	✓	✓	✓	✓	✓	✓
Health Understanding and Skills [n]	• Health literacy	✓							
Healthcare Access, Quality, and Trust [o]	• Age at HIV diagnosis<br>• Healthcare access, satisfaction, provider trust<br>• Insurance coverage	✓							

Core 5 Social Determinants of Health Screening Tool [p]	• Food access, housing, transportation, safety	✓			✓				
Life Experiences Survey [q]	• Stigma, discrimination<br>• HIV disclosure	✓							
General Life Satisfaction [r]	• Quality of life	✓		✓	✓	✓		✓	
Perceived Stress [r]	• Anxiety, stress, coping	✓		✓	✓	✓		✓	
Self-Efficacy [r]	• Confidence in handling challenges	✓		✓	✓	✓		✓	
Emotional Support [r]	• Social support and companionship	✓		✓	✓	✓		✓	
Instrumental Support [r]	• Practical support for completing tasks	✓		✓	✓	✓		✓	
Mental Health	• Prior mental health support	✓							
Postpartum Mental Health [s]	• Postpartum depression symptoms	✓		✓	✓	✓		✓	
Anxiety Screen [t]	• Anxiety symptoms	✓		✓	✓	✓		✓	
Primary Care PTSD Screen [u]	• Post traumatic stress disorder symptoms	✓		✓	✓	✓		✓	
Substance Use [v]	• Use of tobacco, alcohol, other substances<br>• Impact on infant feeding decision	✓			✓			✓	
Intimate Partner Violence Survey [w]	• Verbal, physical, sexual abuse	✓		✓	✓	✓			

Abbreviations: FIMR, Fetal Infant Mortality Review; PTSD, Post-Traumatic Stress Disorder

[a] Survey is only administered to breastfeeding participants

[b] Some questions adapted from the Prenatal Breastfeeding Education Survey (https://www.holzer.org/care-treatment/pregnancy-birth/online-childbirth-class/prenatal-breastfeeding-education-survey/)

[c] Some questions adapted from the 2022 Maternal and Infant Health Assessment, California Dept of Public Health (https://www.cdph.ca.gov/Programs/CFH/DMCAH/MIHA/Pages/Questionnaire.aspx)

[d] Questionnaires obtained from: https://agesandstages.com/

[e] Some questions adapted from:

1. The 2022 Maternal and Infant Health Assessment, California Dept of Public Health (https://www.cdph.ca.gov/Programs/CFH/DMCAH/MIHA/Pages/Questionnaire.aspx)
2. The Infant Feeding Practices Study (IFPS) II Infant Month 2 Questionnaire (https://www.cdc.gov/breastfeeding-data/studies/questionnaires.html)

[f] Decisional Conflict Scale (1)

[g] Survey is only administered to participants who never breastfed

[h] The Breastfeeding Self-Efficacy Scale (2)

[i] The Beginning Breastfeeding Survey - Cumulative (3)

[j] The Maternal Breastfeeding Evaluation Scale (4)

[k] Decision Regret Scale (5)

[l] Some questions adapted from: “The emotional and practical experiences of formula-feeding” (6)

[m] Some questions adapted from: “Three-Item Self-Report Measure for Medication Adherence” (7)

[n] Some questions adapted from: “Brief questions to identify patients with inadequate health literacy” (8)

[o] Some questions adapted from: the “Health Care Relationship Trust Scale” (9)

[p] Core 5 Social Determinants of Health Screening Tool (10)

[q] Some questions adapted from:

1. The Everyday Discrimination Measure (11, 12)
2. Experiences of Discrimination (https://hsph.harvard.edu/research/krieger-group/)
3. The 12-item HIV Stigma Scale (13)
4. HIV Disclosure Status (14)

[r] NIH Toolbox: https://www.healthmeasures.net/explore-measurement-systems/nih-toolbox/intro-to-nih-toolbox/list-of-adult-emotion-measures

[s] Edinburgh Postnatal Depression Scale (15)

[t] Generalized Anxiety Disorder 7-item (GAD-7) (16)

[u] The Primary Care PTSD Screen for DSM-5 (17)

[v] Some questions adapted from: “4Ps Screening Tool” (18)

[w] Universal Violence Prevention Screening Protocol (19)

References for Supplementary Table

1. O'Connor AM. Validation of a decisional conflict scale. Med Decis Making. 1995;15(1):25-30.

2. Dennis CL. The breastfeeding self-efficacy scale: psychometric assessment of the short form. J Obstet Gynecol Neonatal Nurs. 2003;32(6):734-44.

3. Mulder P. Revision of the Beginning Breastfeeding Survey: a cumulative assessment of breastfeeding. J Nurs Meas. 2013;21(1):80-95.

4. Leff EW, Jefferis SC, Gagne MP. The development of the Maternal Breastfeeding Evaluation Scale. J Hum Lact. 1994;10(2):105-11.

5. Brehaut JC, O'Connor AM, Wood TJ, Hack TF, Siminoff L, Gordon E, et al. Validation of a decision regret scale. Med Decis Making. 2003;23(4):281-92.

6. Fallon V, Komninou S, Bennett KM, Halford JCG, Harrold JA. The emotional and practical experiences of formula-feeding mothers. Matern Child Nutr. 2017;13(4).

7. Wilson IB, Lee Y, Michaud J, Fowler FJ, Jr., Rogers WH. Validation of a New Three-Item Self-Report Measure for Medication Adherence. AIDS Behav. 2016;20(11):2700-8.

8. Chew LD, Bradley KA, Boyko EJ. Brief questions to identify patients with inadequate health literacy. Fam Med. 2004;36(8):588-94.

9. Bova C, Fennie KP, Watrous E, Dieckhaus K, Williams AB. The health care relationship (HCR) trust scale: development and psychometric evaluation. Res Nurs Health. 2006;29(5):477-88.

10. Bechtel N, Jones A, Kue J, Ford JL. Evaluation of the core 5 social determinants of health screening tool. Public Health Nurs. 2022;39(2):438-45.

11. Kessler RC, Mickelson KD, Williams DR. The prevalence, distribution, and mental health correlates of perceived discrimination in the United States. J Health Soc Behav. 1999;40(3):208-30.

12. Williams DR, Yan Y, Jackson JS, Anderson NB. Racial Differences in Physical and Mental Health: Socio-economic Status, Stress and Discrimination. J Health Psychol. 1997;2(3):335-51.

13. Reinius M, Wettergren L, Wiklander M, Svedhem V, Ekstrom AM, Eriksson LE. Development of a 12-item short version of the HIV stigma scale. Health Qual Life Outcomes. 2017;15(1):115.

14. Modi RA, McGwin Gl, Jr., Willig JH, Westfall AO, Griffin RL, Amico R, et al. Factors Associated with HIV Disclosure Status Among iENGAGE Cohort of New to HIV Care Patients. AIDS Patient Care STDS. 2020;34(5):213-27.

15. Cox JL, Holden JM, Sagovsky R. Detection of postnatal depression. Development of the 10-item Edinburgh Postnatal Depression Scale. Br J Psychiatry. 1987;150:782-6.

16. Spitzer RL, Kroenke K, Williams JB, Lowe B. A brief measure for assessing generalized anxiety disorder: the GAD-7. Arch Intern Med. 2006;166(10):1092-7.

17. Prins A, Bovin MJ, Smolenski DJ, Marx BP, Kimerling R, Jenkins-Guarnieri MA, et al. The Primary Care PTSD Screen for DSM-5 (PC-PTSD-5): Development and Evaluation Within a Veteran Primary Care Sample. J Gen Intern Med. 2016;31(10):1206-11.

18. Ewing H. A practical guide to intervention in health and social services with pregnant and postpartum addicts and alcoholics: theoretical framework, brief screening tool, key interview

questions, and strategies for referral to recovery resources. . The Born Free Project. 1990;Contra Costa County Department of Health Services.

19. Heron SL, Thompson MP, Jackson E, Kaslow NJ. Do responses to an intimate partner violence screen predict scores on a comprehensive measure of intimate partner violence in low-income black women? Annals of emergency medicine. 2003;42(4):483-91.
